# Input-specific excitation of olfactory cortex microcircuits

**DOI:** 10.3389/fncir.2012.00069

**Published:** 2012-09-19

**Authors:** Victor M. Luna, Alexei Morozov

**Affiliations:** Unit on Behavioral Genetics, National Institute of Mental HealthBethesda, MD, USA

**Keywords:** piriform cortex, interneuron, amygdala, olfaction, emotion, circuit, synapse, optogenetic

## Abstract

Every higher-order association cortex receives a variety of synaptic signals from different regions of the brain. How these cortical networks are capable of differentially responding to these various extrinsic synaptic inputs remains unclear. To address this issue, we studied how the basolateral amygdala (BLA) and the anterior piriform cortex (aPC) were functionally connected to the association olfactory cortex, the posterior piriform cortex (pPC). We infected the BLA and aPC with adeno-associated virus expressing channelrhodopsin-2-Venus fusion protein (ChR2-AAV) and recorded the excitatory postsynaptic currents (EPSC) resulting from photostimulation of either BLA or aPC axons in the major classes of excitatory and inhibitory neurons of the pPC. We found that BLA and aPC axons evoked monosynaptic EPSCs in every type of pPC neuron, but each fiber system preferentially targeted one excitatory and one inhibitory neuronal subtype. BLA fibers were most strongly connected to deep pyramidal cells (DP) and fast-spiking interneurons (FS), while aPC axons formed the strongest synaptic connections with DPs and irregular-spiking interneurons (IR). Overall, our findings show that the pPC differentially responds to amygdaloid versus cortical inputs by utilizing distinct local microcircuits, each defined by one predominant interneuronal subtype: FS for the BLA and IR for the aPC. It would thus seem that preferential excitation of a single neuronal class could be sufficient for the pPC to generate unique electrophysiological outputs in response to divergent synaptic input sources.

## Introduction

Association cortices are inundated with a host of extrinsic synaptic inputs. The unique information contained in these electrical signals depends on the specific area of the brain that originally generated and transmitted them. It stands to reason then that there should be mechanisms in place to help association cortical networks recognize the identity of incoming synaptic inputs based on their anatomical origin. In order to elucidate these mechanisms, it is necessary to understand how specific areas of the brain are functionally connected to particular association cortices.

We studied the functional synaptic connections from the basolateral amygdala (BLA) and anterior piriform cortex (aPC) to the local circuits found in the posterior piriform cortex (pPC). The pPC is a trilaminar higher-order association cortex tasked with assembling various olfactory and non-olfactory synaptic inputs to help form meaningful representations of odor quality (e.g., fruity) as opposed to odorant identity (e.g., lemon) (Gottfried et al., 2006; Kadohisa and Wilson, 2006; Howard et al., 2009). Since the most vivid perceptions of odor quality are essentially shaped by both emotion and sensation, we chose to study the synaptic inputs from the BLA and aPC to the pPC. The BLA processes synaptic information related to emotion and motivation (LeDoux, 2000), while the aPC encodes signals that represent the physical features of odors or odorant identity (Gottfried et al., 2006; Kadohisa and Wilson, 2006).

We combined optogenetic and electrophysiological approaches (also known as “optophysiology”; Hagiwara et al., 2012) to determine how BLA and aPC axonal projections were effectively connected to the major classes of pPC neurons. We used normalized amplitudes of photostimulation-evoked excitatory postsynaptic currents (EPSCs), termed the “nEPSC,” as a measure of functional synaptic connectivity and compared this parameter across different pPC cell types. We were then able to definitively identify the primary pPC neuronal types BLA and aPC selectively excited. Our findings show that the pPC could differentially respond to BLA and aPC synaptic signals by preferential excitation of a single pPC neuronal subtype. This target-specific mechanism may be a necessary first step that allows the pPC to extract the content of each extrinsic synaptic input it receives before systematically combining them into unified olfactory representations.

## Materials and methods

### ChR2-AAV infection

**Figure 1:**
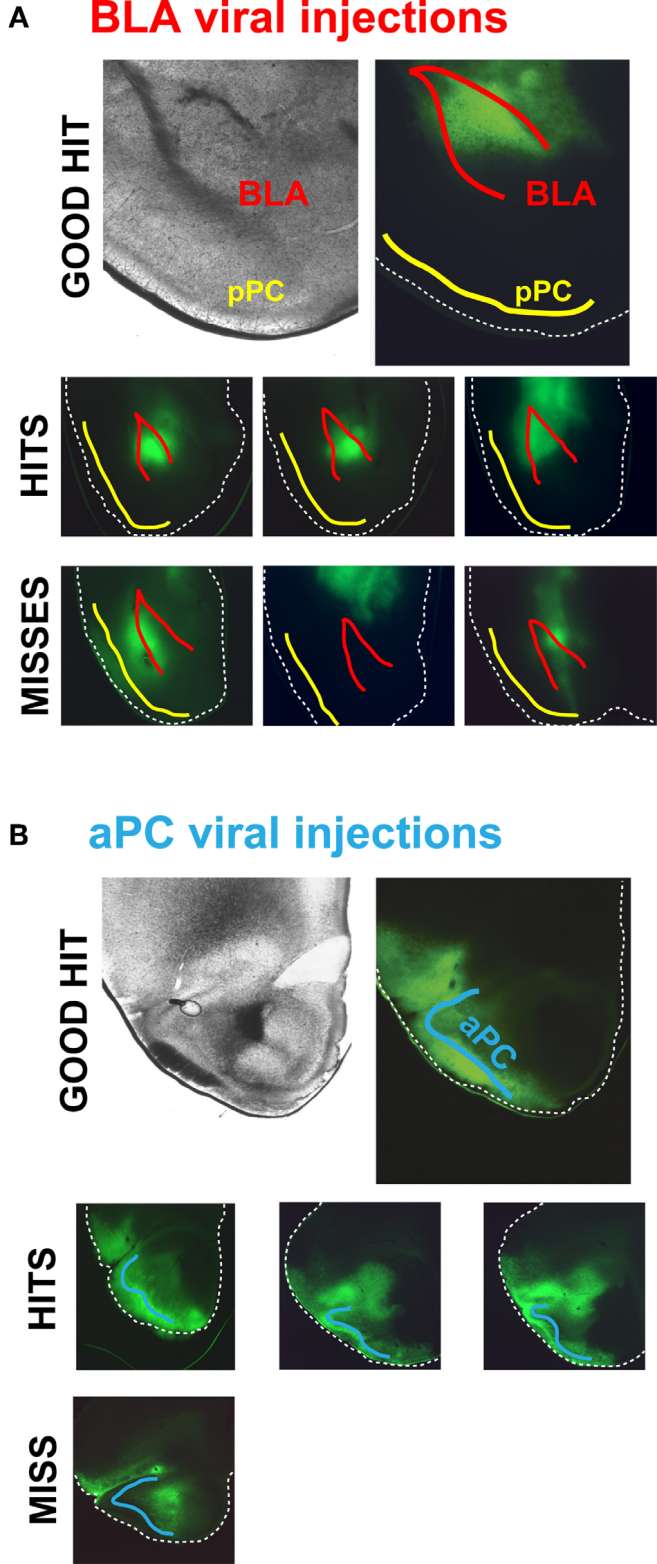
**ChR2-AAV injections in and outside of the BLA and aPC. (A)** *Top row*, An example of an ideal BLA injection. *Middle row*, Examples of common on-target BLA injections in different mouse brains. ChR2-AAV was specifically infected in the BLA in ~25% of the injected mice. *Bottom row*, Examples of the most common off-target BLA injections. These mouse brains were not used for experiments. **(B)** *Top row*, An example of an ideal ChR2-AAV injection in the aPC. *Middle row*, Examples of typical on-target aPC injections in different mouse brains. The aPC was successfully infected with ChR2-AAV in ~90% of injected mice. *Bottom row*, An example of the rare off-target aPC injection.

Surgeries were performed according to NIMH ACUC-approved protocols. ChR2-AAV pseudo type 1 virus was prepared at the titer of 10^12^ viral particles per ml by UNC Gene Therapy Vector Core (Chapel Hill, NC) using Addgene plasmid 20071 (Petreanu et al., 2009). 25- to 30-day old 129SvEv/C57BL/6J F1 hybrid or GAD67-GFP (Tamamaki et al., 2003) male mice on 129SvEv/C57BL/6J mixed background were injected bilaterally with 0.5 μl of virus in: (1) BLA—1.6 mm posterior from bregma, 3.2 mm lateral from bregma, and 3.6 mm ventral from brain surface, or (2) aPC—1.8 mm anterior from bregma, 2.2 mm lateral from bregma, and 3.7 mm ventral from brain surface. The BLA was specifically hit in only 25% of all mouse injections; we therefore assessed the BLA injections in all animals before optophysiological experiments (Figure 1A). We had a much higher success rate when injecting the aPC, a 90% hit rate, which was determined in a subset of animals (Figure 1B).

**Figure 2:**
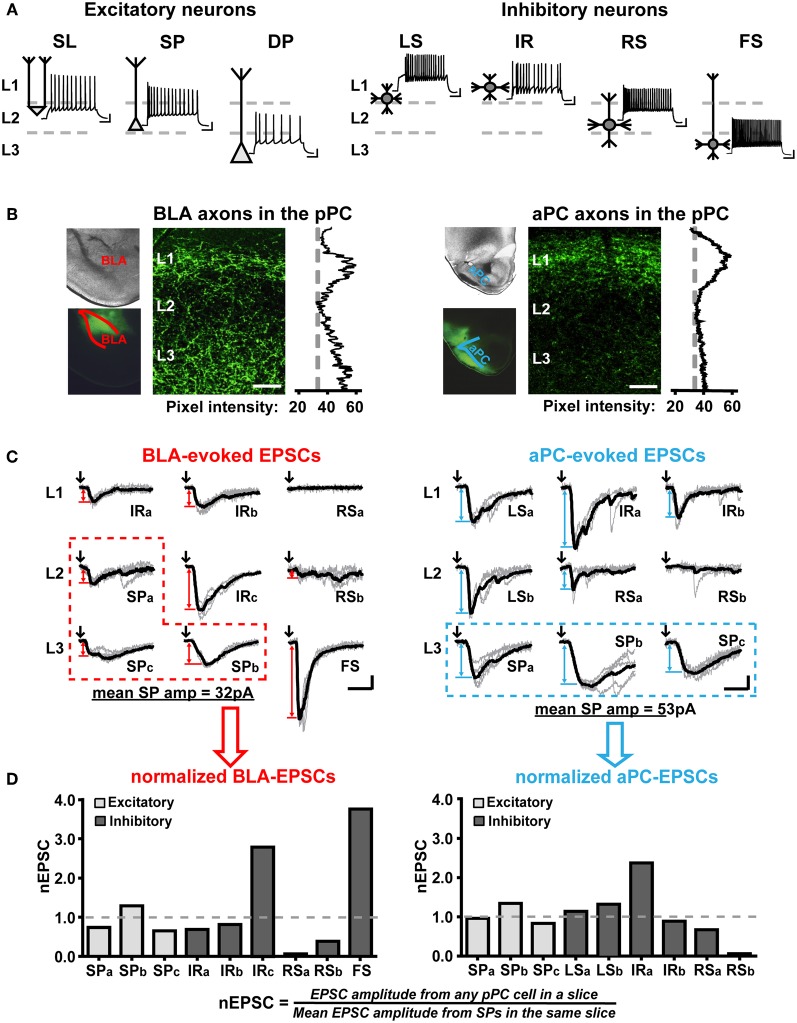
**Qualitative and quantitative characterizations of functional BLA and aPC synaptic connectivity onto the major pPC neuronal classes. (A)** Action potential firing patterns and the most common somatic and dendritic locations of the major pPC neuronal classes. Semilunar cell (SL); superficial pyramidal cell (SP); deep pyramidal cells (DP); late-spiking interneuron (LS); irregular-spiking interneuron (IR); regular-spiking interneuron (RS); fast-spiking interneuron (FS). Scale bars represent 20 mV and 100 ms. **(B)** Images showing axonal fibers entering two different pPC brain slices from a ChR2-AAV-infected BLA and aPC. Pixel intensity plots highlight the differences in BLA and aPC innervation patterns in the pPC. 50 μm scale bar. **(C)** Examples of BLA- and aPC-evoked EPSCs (*V*_*m*_ = −75 mV) recorded from randomly chosen neurons in a single BLA-infected coronal pPC slice (left) and a single aPC-infected coronal pPC slice (right). Neurons belonging to the same class were distinguished from one another using lower-case letters (e.g., IRa and IRb are two different IR cells in the same slice). Raw traces (5 per cell) are in gray; the average EPSCs from these traces are shown in black. Only these black EPSC traces were used to quantify BLA or aPC synaptic connectivity. Black arrows indicate the onset of 1 ms light pulses. Red (BLA) and blue (aPC) double-arrows indicate how EPSC peak amplitude was measured. Red (BLA) and blue (aPC) dashed boxes highlight the EPSCs from SPs that will be used to normalize all other EPSCs in the slice. Scale bars represent 20 pA and 10 ms. **(D)** Plots showing the EPSCs in **(C)** were converted to nEPSCs to enable comparisons of BLA-to-pPC and aPC-to-pPC synaptic connectivity.

To assess the axonal projection patterns of the BLA or aPC to the pPC (Figure 2B), mice were intracardially perfused with 4% paraformaldehyde (PFA) at least 30 days after surgery. Brains were then post-fixed in PFA overnight and sliced into 100 μm sections. Low magnification images of the injection area were obtained using Leica DMRB fluorescence microscope (Leica, Bannockburn, IL) equipped with CoolSnap CCD camera (Photometrics, Tucson, AZ). High magnification images of ChR2-AAV-infected BLA or aPC fibers innervating the pPC were obtained using Zeiss LSM510 confocal microscope (Carl Zeiss, Thornwood, NY) and analyzed using ImageJ software (National Institutes of Health, USA).

### Electrophysiology

Electrophysiology experiments were performed at least 30 days after ChR2-AAV infection in either the BLA or aPC. 300 μm-thick pPC coronal brain slices were made using procedures modified from those previously described for the preparation of amygdala slices (Tsvetkov et al., 2002). Slices were cut using DSK Microslicer (Ted Pella, Redding, CA) in ice cold partial sucrose artificial cerebrospinal fluid (ACSF) solution containing (in mM): 80 NaCl, 3.5 KCl, 4.5 MgSO_4_, 0.5 CaCl_2_, 1.25 H_2_PO_4_, 25 NaHCO_3_, 10 glucose, and 90 sucrose equilibrated with 95% O_2_/5% CO_2_ and stored in the same solution at room temperature for at least 45 min before recording (Daw et al., 2009). Slices were transferred to a recording chamber superfused at 1 ml/min with ACSF equilibrated with 95% O_2_/5% CO_2_ and containing (in mM): 119 NaCl, 2.5 KCl, 1 MgSO_4_, 2.5 CaCl_2_, 1.25 H_2_PO_4_, 26 NaHCO_3_, 10 glucose, pH 7.4. Temperature was maintained at 29 ± 1°C. Patch pipettes (4–6 MΩ) were filled with (in mM): 120 K-gluconate, 5 NaCl, 1 MgCl_2_, 10 HEPES, and 0.2 EGTA, 2 ATP-Mg, 0.1 GTP-Na, pH 7.3.

For optophysiological experiments, 470 nm light pulses (10.8 mW) were generated using an LED lamp and driver (Thorlabs, Newton, NJ) and delivered through a 40× objective in an Axioskop FS 2 (Carl Zeiss, Thornwood, NY). Single light pulses (1 ms duration) delivered every 15–20 s were used to activate BLA or aPC fibers in pPC brain slices while recording evoked monosynaptic EPSCs (<5 ms current onset latency) in randomly chosen pPC neurons. These cells were visualized using differential interference optics and identified based on previously characterized passive electrical properties and action potential firing patterns (Figure 2A; Tseng and Haberly, 1989; Suzuki and Bekkers, 2006, 2011, 2012; Young and Sun, 2009; Wiegand et al., 2011).

Current and voltage signals were recorded with a MultiClamp 700 B amplifier (Molecular Devices, USA), digitized at 5–10 kHz, and filtered at 2.5–4 kHz. Data were acquired and analyzed using Axograph (Axograph Scientific, Sydney, Australia).

Statistical analyses were done using Student's *t*-test (for 2 groups) or between-groups ANOVA (for 3 or more groups) with *post-hoc* comparisons conducted using Tukey's HSD test. All significance tests were two-tailed with α = 0.05.

### Identifying major pPC neuronal classes

We used GAD67-GFP mice to help locate inhibitory cells in pPC coronal slices. We also distinguished inhibitory from excitatory cells based on their threshold spike peak amplitudes (≤60 mV for inhibitory and (≥80 mV for excitatory cells). We then used action potential firing patterns in response to 500 ms currents steps to determine the specific inhibitory or excitatory subtype of a cell (Figure 2A).

Briefly, we identified subtypes of excitatory neurons (Figure 2A) based on the location of their soma, initial spike burst, and input resistance (Tseng and Haberly, 1989; Suzuki and Bekkers, 2006, 2011; Wiegand et al., 2011). Superficial pyramidal cells (SP) were: located in the deeper part of L2 and the superficial regions of L3, fired an initial high-frequency burst of spikes at current step onset, and had input resistances ranging from 150–200 MΩ. Deep pyramidal cells (DP) were: located in the deeper regions of L3, fired an initial burst of spikes, and had low input resistances ranging from 50–100 MΩ. Semilunar cells (SL) were: located in the superficial parts of L2, did not fire instantaneous high-frequency burst of spikes at step onset, and had high input resistances comparable to interneurons (220–250 MΩ) but threshold spike peak amplitudes in between interneurons and excitatory cells (~70 mV).

We differentiated the various types of pPC interneurons from each other (Figure 2A) based on the classification system described by Young and Sun (2009). Late-spiking interneurons (LS) were identified by their characteristic delayed spike onset when stimulated at near-threshold current intensities. Irregular-spiking interneurons (IR) demonstrated highly variable, almost stuttering, spike patterns at near-threshold levels. Fast-spiking interneuron (FS) had spike half-widths <1 ms and maximum steady-state frequencies of ~100 Hz. Inhibitory cells that did not fall under these three classifications were considered regular-spiking interneurons (RS). We also recorded from a very small number of low-threshold spiking cells (LTS), however due to their scarcity (Young and Sun, 2009) and similarity to RS, we grouped these interneurons with RS as had been previously recommended (Suzuki and Bekkers, 2010).

## Results

As in previous studies, we found that BLA axons project to Layer 1 (L1) and L3 of the pPC while aPC axons primarily coalesce in L1 (Figure 2B; Haberly and Price, 1978; Majak et al., 2004; Franks et al., 2011; Hagiwara et al., 2012). Since most pPC neurons send dendritic arbors to L1 and L3 (Figure 2A; Haberly, 1983; Tseng and Haberly, 1989; Ekstrand et al., 2001; Suzuki and Bekkers, 2006; Young and Sun, 2009; Gavrilovici et al., 2010; Wiegand et al., 2011), it could be expected that BLA and aPC fibers would form functional synapses with most, if not all, the major classes of pPC neurons. However, it is unlikely that BLA or aPC synaptic connections would be homogeneous across the anatomically and biophysically diverse array of pPC neuronal subtypes. As such, it would be expected that perhaps certain neuronal classes would receive significantly greater—or lesser—excitatory currents over others. In addition, since only the BLA projects to L3 (Figure 2B), it could also be predicted that the BLA and aPC would preferentially excite different neuronal components of the pPC network.

### Measuring functional synaptic connectivity

To be able to assess if the BLA or aPC does exert discriminatory excitation over select types of pPC neurons, we needed to be able to measure synaptic currents evoked by either fiber system across the major classes of pPC neurons (Figure 2A). To accomplish this, we first infected BLAs or aPCs with ChR2-AAV (Figures 1, 2B). We then photostimulated BLA or aPC axons in pPC coronal slices (Figure 2B) and recorded the excitatory postsynaptic currents (EPSCs) evoked in neurons connected to either fiber system using somatic whole-cell recordings (*V*_*m*_ = −75 mV) (Figure 2C). We restricted our recordings to coronal brain slices located ~1.5–1.7 mm posterior to bregma (Figure 1A; based on Franklin and Paxinos, 2008). We measured only monosynaptic EPSCs (≤5 ms current onset latency; Figure 2C) to assess solely the impact of BLA or aPC fibers, avoiding complications from pPC recurrent excitatory circuits (Haberly and Price, 1978; Franks et al., 2011; Hagiwara et al., 2012). These monosynaptic currents were also the largest EPSCs evoked after each light pulse and thus represented the peak current response of each recorded pPC cell (Figure 2C). We used these EPSCs as an initial assay for BLA or aPC connective strength onto different pPC neuronal classes.

For each brain slice, we recorded from randomly chosen neurons across the three layers of the pPC (Figure 2A). As much as possible, we used GAD67-GFP mice to aid in identification of interneurons; however, we relied mostly on passive electrical properties and action potential firing patterns to identify pPC neuronal classes (Figure 2A; see “Materials and Methods”). As shown in the examples in Figure 2C, it would appear that within a single pPC slice, different pPC neuronal types (Figure 2A) received different magnitudes of excitation from the BLA or aPC. BLA synaptic inputs evoked the largest EPSCs in a FS whose soma was located in L3 and no EPSCs in a RS located in L1, designated as “RSa” to differentiate it from other RS in the same slice (Figure 2C). On the other hand, aPC fibers evoked the largest EPSCs in an IR in L1 (“IRa”), while a RS in L2 (“RSb”) received no excitatory currents (Figure 2C). However, to more accurately characterize BLA-to-pPC and aPC-to-pPC connectivity, we needed to evaluate BLA- and aPC-driven pPC excitation patterns in a larger number of neurons across multiple pPC slices.

Unfortunately, there is a fair amount of variability in EPSC amplitudes between pPC slice preparations that simply arises from possible differences in the levels of ChR2-AAV infection among mice (Figure 1; Franks et al., 2011; Hagiwara et al., 2012). It was therefore not feasible to just use EPSC amplitude as a measure of connectivity for populations of neurons across various pPC slices. We instead used normalized EPSCs (“nEPSC”) calculated by dividing the EPSC amplitude in a given cell by the mean EPSC recorded from 3 to 5 SP in the same brain slice (Figures 2C,D). We chose to normalize using EPSCs in SP because these neurons are the easiest to locate and identify (Figure 2A) and because there is, on average, very little variability in EPSC amplitudes among SPs within any given slice (mean ± SEM; CV_BLA−EPSCs_ = 0.41 ± 0.08, *n* = 11 slices, 3–5 cells in each; CV_aPC−EPSCs_ = 0.43 ± 0.05, *n* = 10 slices, 3–5 cells in each).

The use of nEPSCs controls for artifactual variations in EPSC magnitude and can thus be used as a final measure of functional synaptic connectivity within pPC slice preparations. By converting EPSCs (Figure 2C) to nEPSCs (Figure 2D), we were able to generate nEPSC plots that more quantitatively represented the magnitude variations in BLA- and aPC-driven excitation across pPC neuronal classes in a single slice. More importantly, however, is that we can use these nEPSC plots to assess BLA and aPC connectivity in neuronal populations found across a number of pPC slices. We could then more accurately identify the pPC neuronal components preferentially recruited—or not—by BLA and aPC synaptic afferents.

### Determining BLA- and aPC-driven pPC microcircuits

**Figure 3:**
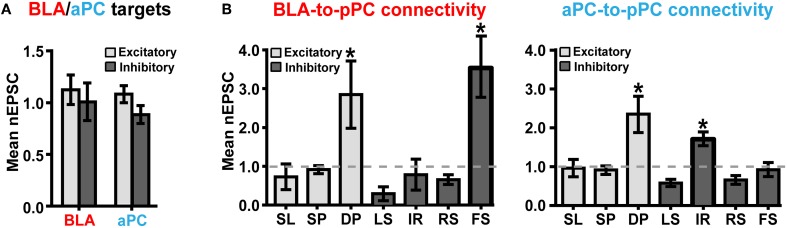
**BLA and aPC preferentially recruit DP + FS and DP + IR microcircuits, respectively. (A)** Histograms showing that, in general, the strengths of BLA and aPC synaptic connections (or nEPSCs) onto pPC excitatory and inhibitory neurons do not differ. (B) Cumulative nEPSC plots generated from neurons in multiple brain slices. Notice that that the strengths of BLA and aPC synaptic connections are strikingly similar across nearly all pPC neuronal classes. The only significant difference between the BLA and aPC fiber systems lies in their functional synaptic connections onto FS and IR. Stars (^*^) indicate that the mean nEPSC of a pPC neuronal type was significantly (*P* < 0.0001) different than the nEPSC of SPs (~1.0). Error bars represent s.e.m.

We used nEPSC as our primary measure of functional BLA or aPC connective strength. We were thus able to generate cumulative nEPSC plots for every major pPC neuronal class found in multiple brain slices in order to determine BLA-to-pPC and aPC-to-pPC connectivity. As predicted, BLA and aPC axons, on average, excited all types of pPC neurons (Figure 3). Surprisingly, we found equivalent excitation of pPC excitatory (E) and inhibitory (I) neurons from both the BLA (*E*_nEPSC_ = 1.12 ± 0.14, *n* = 55 cells; *I*_nEPSC_ = 1.01 ± 0.18, *n* = 50 cells; 17 slices; *P* = 0.6142) and aPC (*E*_nEPSC_ = 1.08 ± 0.08, *n* = 48; *I*_nEPSC_ = 0.88 ± 0.09, *n* = 54; 18 slices; *P* = 0.1037) (Figure 3A).

**Figure 4:**
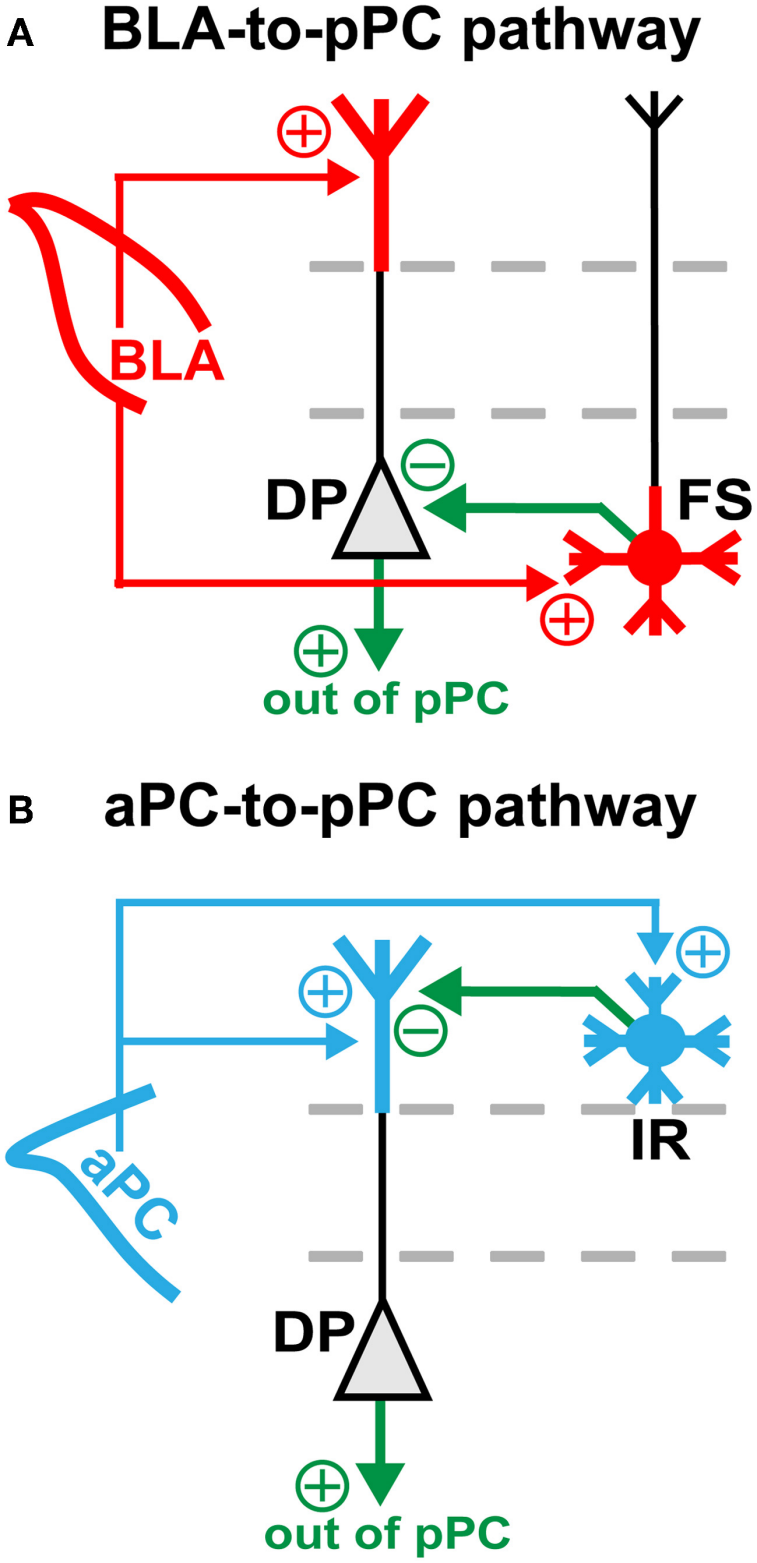
**BLA- and aPC-dependent feed-forward inhibition in the pPC.** Diagrams showing the proposed **(A)** BLA- and **(B)** aPC-to-pPC functional connective schemes. Red and blue arrows represent BLA and aPC axonal projections, respectively. Green arrows represent axons emanating from local pPC excitatory (triangle) or inhibitory (circle) neurons. Plus (+) and minus (−) signs indicate whether axons are excitatory or inhibitory. Note that the BLA drives feed-forward somatic inhibition, while the aPC drives feed-forward dendritic inhibition of pPC pyramidal cells—DPs and SPs alike.

However, it was also apparent that specific pPC neuronal subtypes had significantly larger BLA-nEPSCs and aPC-nEPSCs than other neuronal classes [BLA: *F*_(6, 95)_ = 9.155, *P* < 0.0001; aPC: *F*_(6, 95)_ = 11.86, *P* < 0.0001; Figure 3B]. *Post-hoc* comparisons conducted using Tukey's HSD test revealed that for BLA inputs (Figure 3B), only DP (mean nEPSC = 2.85 ± 0.87, *n* = 11) and FS (nEPSC = 3.53 ± 0.75, *n* = 6) had significantly different nEPSCs from SP (nEPSC = 0.92 ± 0.10, *n* = 37) (*P* < 0.0001). All other neuronal subtypes had similar magnitude nEPSCs to SP_nEPSC_ [*F*_(4, 82)_ = 1.412, *P* = 0.2372] (Figure 3B). FS are involved in somatic inhibition of pyramidal cells (Young and Sun, 2009; Stokes and Isaacson, 2010; Suzuki and Bekkers, 2012), while DPs are involved in local recurrent excitation of the deeper portions of the pPC as well as in sending long-range outputs to structures like the amygdala, entorhinal cortex, and prefrontal cortex (Figure 4A; Tseng and Haberly, 1989; Chen et al., 2003).

Interestingly, even though majority of aPC axons were restricted to L1 of the pPC, the aPC still preferentially excited DP (nEPSC = 2.36 ± 0.47, *n* = 8); but instead of FS, the aPC targeted IR (nEPSC = 1.61 ± 0.17, *n* = 13) (Figure 3B; SP_nEPSC_ = 0.91 ± 0.07, *n* = 37; *P* < 0.0001 for DP and FS). Like the BLA, the aPC was also connected to other pPC neuronal subtypes to a smaller yet similar extent [*F*_(4, 77)_ = 2.034, *P* = 0.0979] (Figure 3B). IR, unlike FS, are primarily involved in dendritic inhibition of pyramidal cells (Figure 4B; Young and Sun, 2009; Stokes and Isaacson, 2010; Suzuki and Bekkers, 2012). These findings therefore demonstrate that the pPC is perhaps capable of discriminating BLA from aPC synaptic information by utilizing biophysically distinct microcircuits, each distinguished by a single interneuronal subtype: DP + FS for BLA and DP + IR for aPC. Given the fast onset of BLA- and aPC-evoked peak current responses (≤5 ms; Figure 2C), it then seems theoretically possible that the pPC could near-instantaneously encode different types of synaptic inputs from other cortical and sub-cortical structures using only a limited set of neuronal subtypes.

## Discussion

### Advantages and limitations of pPC neural circuit analysis

Previous studies in the cerebral cortex have already shown that, depending on their anatomical origin, extrinsic axons preferentially target a select subset of neuronal subtypes within a given neural network (Freund and Gulyas, 1991; Hornung and Celio, 1992; Staiger et al., 1996). However, actual electrical signals were not measured in these studies and, as such, functional synaptic connectivity could not be thoroughly evaluated. We resolved this problem by combining electrophysiology with optogenetics to isolate axons coming only from the BLA or aPC and recording the EPSCs resulting from their activation in the pPC.

We essentially expanded on the optophysiological approaches previously used in olfactory cortex circuit analysis (Franks et al., 2011; Hagiwara et al., 2012) by measuring functional synaptic connections not just in a single cell type—past studies used only pyramidal cells—but on an array of pPC neuronal classes, excitatory and inhibitory alike. We developed a normalized measure of effective synaptic strength, the nEPSC, to compare functional BLA or aPC synaptic connections in every major pPC neuronal type within and across brain slice preparations. By assessing nEPSCs, we found that the BLA and aPC preferentially excite distinct pPC microcircuits, each defined by the same excitatory neuron, yet distinguished by their interneuronal components: DP + FS for the BLA and DP + IR for the aPC. This microcircuit-based mechanism may be an important first step that allows the pPC to differentially encode divergent types of synaptic information emanating from the BLA and aPC.

A major limitation of our studies was that our identification of BLA- and aPC-driven pPC microcircuits was fully dependent on classifications based on the passive and active biophysical properties of pPC neurons (Figure 2A; see “Materials and Methods”; Tseng and Haberly, 1989; Suzuki and Bekkers, 2006, 2011; Young and Sun, 2009; Wiegand et al., 2011). It is entirely possible that more specialized pPC neuronal subclasses could be characterized in the future, perhaps by combining both biophysical and immunohistochemical criteria (Ekstrand et al., 2001; Gavrilovici et al., 2010). We would then have to re-examine the identities of the microcircuits we elucidated to factor in these new categories. However, it is also possible that the emergence of new neuronal subtypes may not drastically alter the BLA-to-pPC and aPC-to-pPC connective pathways we deciphered. This is because the broad neuronal classes we used have been shown to be fairly ubiquitous and reliable in classifying neurons across various other cortical structures (Connors and Gutnick, 1990; Kawaguchi, 1993, 1995; Cauli et al., 1997; Chu et al., 2003; Young and Sun, 2009).

Additionally, our studies focused only on the initial monosynaptic excitation of pPC neurons in response to isolated BLA and aPC afferents. We chose this small temporal window because this is the only time when we could accurately measure pure BLA and aPC synaptic inputs unaltered by the strong intrinsic circuitry of the pPC (Haberly and Price, 1978; Franks et al., 2011; Hagiwara et al., 2012). However, further studies need to be undertaken to assess how BLA- and aPC-driven pPC microcircuits change over time as a result of their dynamic interaction with the recurrent circuits of the pPC and perhaps with other extrinsic input sources as well. Other limitations of using optophysiological approaches for neural circuit analysis (see also Franks et al., 2011; Hagiwara et al., 2012) pertain to the possibilities that the ChR2-AAV may be preferentially infected only in a subset of BLA or aPC projection neurons and that there may be regional differences in BLA and aPC connectivity across the the pPC. Unfortunately, we were unable to fully rule out any of these potential scenarios.

### Functional implications of BLA-to-pPC and aPC-to-pPC connectivity

Our results allow for specific predictions on how the BLA and aPC could near instantaneously (≤5 ms) impact pPC functioning as a result of their preferential synaptic connectivity onto a restricted set of pPC neurons. Our findings suggest that the BLA, via FS, controls pPC spike outputs by feed-forward somatic inhibition of pyramidal cells (Figure 4A; Young and Sun, 2009; Stokes and Isaacson, 2010; Suzuki and Bekkers, 2012). The BLA can thus rapidly shift cortical activity in response to imminent dangers or rewards or other emotionally-charged cues (LeDoux, 2000). In contrast, the aPC, via IR, regulates the strength of extrinsic and intrinsic synaptic inputs by feed-forward dendritic inhibition of pyramidal cells (Figure 4B; Kanter et al., 1996; Young and Sun, 2009; Stokes and Isaacson, 2010; Suzuki and Bekkers, 2012). This fine synaptic control could be used to dynamically alter synaptic signals related to physical odor features in order to shape perceptions of odor quality or to generate meaningful odor memories (Kanter et al., 1996; Gottfried et al., 2006; Kadohisa and Wilson, 2006; Howard et al., 2009). Additionally, since both the BLA and aPC preferentially excite DP, changes in on-going synaptic information processing as a result of the two different types of feed-forward inhibition can immediately be spread within and out of the pPC through local DP axonal collaterals and long-range DP fiber projections (Figure 4; Tseng and Haberly, 1989; Chen et al., 2003).

In addition, our findings provide important insights not only on how different extrinsic synaptic inputs could be discriminated from one another in an association cortex, but also on how brain regions could perhaps communicate each other's unique synaptic information. That is, through a distinct syntax based on targeted microcircuit excitation (Figure 4). Surprisingly, the neuronal components of the BLA- and aPC-driven pPC microcircuits we identified were remarkably the same, differing only in one interneuron subtype (Figure 3). Thus it appears that the specific action of a single pPC neuronal subtype could be sufficient to distinguish BLA from aPC synaptic inputs. It would then be interesting to see if other types of afferent-driven pPC microcircuits could also be differentiated from one another based on the synaptic predominance of a very restricted number of neuronal subtypes.

### Conflict of interest statement

The authors declare that the research was conducted in the absence of any commercial or financial relationships that could be construed as a potential conflict of interest.

## References

[B1] Cauli B. Audinat E. Lambolez B. Angulo M. C. Ropert N. Tsuzuki K. Hestrin S. Rossier J. (1997). Molecular and physiological diversity of cortical nonpyramidal cells. J. Neurosci. 17, 3894–3906 9133407 10.1523/JNEUROSCI.17-10-03894.1997 PMC6573690

[B2] Chen S. Murakami K. Oda S. Kishi K. (2003). Quantitative analysis of axon collaterals of single cells in layer III of the piriform cortex of the guinea pig. J. Comp. Neurol. 465, 455–465 10.1002/cne.10844 12966568

[B3] Chu Z. Galarreta M. Hestrin S. (2003). Synaptic interactions of late-spiking neocortical neurons in layer 1. J. Neurosci. 23, 96–102 12514205 10.1523/JNEUROSCI.23-01-00096.2003 PMC6742162

[B4] Connors B. W. Gutnick M. J. (1990). Intrinsic firing patterns of diverse neocortical neurons. Trends Neurosci. 13, 99–104 10.1016/0166-2236(90)90185-D 1691879

[B5] Daw M. I. Tricoire L. Erdelyi F. Szabo G. McBain C. J. (2009). Asynchronous transmitter release from cholecystokinin-containing inhibitory interneurons is widespread and target-cell independent. J. Neurosci. 29, 11112–11122 10.1523/JNEUROSCI.5760-08.2009 19741117 PMC2762613

[B6] Ekstrand J. J. Domroese M. E. Feig S. L. Illig K. R. Haberly L. B. (2001). Immunocytochemical analysis of basket cells in rat piriform cortex. J. Comp. Neurol. 434, 308–328 11331531

[B7] Franklin K. B. J. Paxinos G. (2008). The Mouse Brain in Stereotaxic Coordinates. New York, NY: Academic Press.

[B8] Franks K. M. Russo M. J. Sosulski D. L. Mulligan A. A. Siegelbaum S. A. Axel R. (2011). Recurrent circuitry dynamically shapes the activation of piriform cortex. Neuron 72, 49–56 10.1016/j.neuron.2011.08.020 21982368 PMC3219421

[B9] Freund T. F. Gulyas A. I. (1991). GABAergic interneurons containing calbindin D28K or somatostatin are major targets of GABAergic basal forebrain afferents in the rat neocortex. J. Comp. Neurol. 314, 187–199 10.1002/cne.903140117 1686776

[B10] Gavrilovici C. D'Alfonso S. Poulter M. O. (2010). Diverse interneuron populations have highly specific interconnectivity in the rat piriform cortex. J. Comp. Neurol. 518, 1570–1588 10.1002/cne.22291 20187146

[B11] Gottfried J. A. Winston J. S. Dolan R. J. (2006). Dissociable codes of odor quality and odorant structure in human piriform cortex. Neuron 49, 467–479 10.1016/j.neuron.2006.01.007 16446149

[B12] Haberly L. B. (1983). Structure of the piriform cortex of the opossum. I. Description of neuron types with Golgi methods. J. Comp. Neurol. 213, 163–187 10.1002/cne.902130205 6841668

[B13] Haberly L. B. Price J. L. (1978). Association and commissural fiber systems of the olfactory cortex of the rat. J. Comp. Neurol. 178, 711–740 10.1002/cne.901780408 632378

[B14] Hagiwara A. Pal S. K. Sato T. F. Wienisch M. Murthy V. N. (2012). Optophysiological analysis of associational circuits in the olfactory cortex. Front. Neural Circuits 6:18 10.3389/fncir.2012.00018 22529781 PMC3329886

[B15] Hornung J. P. Celio M. R. (1992). The selective innervation by serotoninergic axons of calbindin-containing interneurons in the neocortex and hippocampus of the marmoset. J. Comp. Neurol. 320, 457–467 10.1002/cne.903200404 1629398

[B16] Howard J. D. Plailly J. Grueschow M. Haynes J. D. Gottfried J. A. (2009). Odor quality coding and categorization in human posterior piriform cortex. Nat. Neurosci. 12, 932–938 10.1038/nn.2324 19483688 PMC2834563

[B17] Kadohisa M. Wilson D. A. (2006). Separate encoding of identity and similarity of complex familiar odors in piriform cortex. Proc. Natl. Acad. Sci. U.S.A. 103, 15206–15211 10.1073/pnas.0604313103 17005727 PMC1622801

[B18] Kanter E. D. Kapur A. Haberly L. B. (1996). A dendritic GABAA-mediated IPSP regulates facilitation of NMDA-mediated responses to burst stimulation of afferent fibers in piriform cortex. J. Neurosci. 16, 307–312 8613796 10.1523/JNEUROSCI.16-01-00307.1996 PMC6578711

[B19] Kawaguchi Y. (1993). Groupings of nonpyramidal and pyramidal cells with specific physiological and morphological characteristics in rat frontal cortex. J. Neurophysiol. 69, 416–431 8459275 10.1152/jn.1993.69.2.416

[B20] Kawaguchi Y. (1995). Physiological subgroups of nonpyramidal cells with specific morphological characteristics in layer II/III of rat frontal cortex. J. Neurosci. 15, 2638–2655 7722619 10.1523/JNEUROSCI.15-04-02638.1995 PMC6577784

[B21] LeDoux J. E. (2000). Emotion circuits in the brain. Annu. Rev. Neurosci. 23, 155–184 10.1146/annurev.neuro.23.1.155 10845062

[B22] Majak K. Ronkko S. Kemppainen S. Pitkanen A. (2004). Projections from the amygdaloid complex to the piriform cortex: a PHA-L study in the rat. J. Comp. Neurol. 476, 414–428 10.1002/cne.20233 15282713

[B23] Petreanu L. Mao T. Sternson S. M. Svoboda K. (2009). The subcellular organization of neocortical excitatory connections. Nature 457, 1142–1145 10.1038/nature07709 19151697 PMC2745650

[B24] Staiger J. F. Zilles K. Freund T. F. (1996) Distribution of GABAergic elements postsynaptic to ventroposteromedial thalamic projections in layer IV of rat barrel cortex. Eur. J. Neurosci. 8, 2273–2285 8950092 10.1111/j.1460-9568.1996.tb01191.x

[B25] Stokes C. C. Isaacson J. S. (2010). From dendrite to soma: dynamic routing of inhibition by complementary interneuron microcircuits in olfactory cortex. Neuron 67, 452–465 10.1016/j.neuron.2010.06.029 20696382 PMC2922014

[B26] Suzuki N. Bekkers J. M. (2006). Neural coding by two classes of principal cells in the mouse piriform cortex. J. Neurosci. 26, 11938–11947 10.1523/JNEUROSCI.3473-06.2006 17108168 PMC6674875

[B27] Suzuki N. Bekkers J. M. (2010). Distinctive classes of GABAergic interneurons provide layer-specific phasic inhibition in the anterior piriform cortex. Cereb. Cortex 20, 2971–2984 10.1093/cercor/bhq046 20457693 PMC2978245

[B28] Suzuki N. Bekkers J. M. (2011). Two layers of synaptic processing by principal neurons in piriform cortex. J. Neurosci. 31, 2156–2166 10.1523/JNEUROSCI.5430-10.2011 21307252 PMC6633060

[B29] Suzuki N. Bekkers J. M. (2012). Microcircuits mediating feedforward and feedback synaptic inhibition in the piriform cortex. J. Neurosci. 32, 919–931 10.1523/JNEUROSCI.4112-11.2012 22262890 PMC6621151

[B30] Tamamaki N. Yanagawa Y. Tomioka R. Miyazaki J. Obata K. Kaneko T. (2003). Green fluorescent protein expression and colocalization with calretinin, parvalbumin, and somatostatin in the GAD67-GFP knock-in mouse. J. Comp. Neurol. 467, 60–79 10.1016/j.neures.2004.10.005 14574680

[B31] Tseng G. F. Haberly L. B. (1989). Deep neurons in piriform cortex. I. Morphology and synaptically evoked responses including a unique high-amplitude paired shock facilitation. J. Neurophysiol. 62, 369–385 2769336 10.1152/jn.1989.62.2.369

[B32] Tsvetkov E. Carlezon W. A. Benes F. M. Kandel E. R. Bolshakov V. Y. (2002). Fear conditioning occludes LTP-induced presynaptic enhancement of synaptic transmission in the cortical pathway to the lateral amygdala. Neuron 34, 289–300 10.1016/S0896-6273(02)00645-1 11970870

[B33] Wiegand H. F. Beed P. Bendels M. H. Leibold C. Schmitz D. Johenning F. W. (2011). Complementary sensory and associative microcircuitry in primary olfactory cortex. J. Neurosci. 31, 12149–12158 10.1523/JNEUROSCI.0285-11.2011 21865457 PMC6623216

[B34] Young A. Sun Q. Q. (2009). GABAergic inhibitory interneurons in the posterior piriform cortex of the GAD67-GFP mouse. Cereb. Cortex 19, 3011–3029 10.1093/cercor/bhp072 19359350 PMC2774400

